# 2-Methyl-1-(3-methyl­phenyl­sulfon­yl)naphtho­[2,1-*b*]furan

**DOI:** 10.1107/S1600536814005157

**Published:** 2014-03-12

**Authors:** Hong Dae Choi, Pil Ja Seo, Uk Lee

**Affiliations:** a Department of Chemistry, Dongeui University, San 24 Kaya-dong, Busanjin-gu, Busan 614-714, Republic of Korea; b Department of Chemistry, Pukyong National University, 599-1 Daeyeon 3-dong, Nam-gu, Busan 608-737, Republic of Korea

## Abstract

In the title compound, C_20_H_16_O_3_S, the dihedral angle between the mean planes of the naphtho­furan and 3-methyl­phenyl fragments is 88.56 (2)°. In the crystal, mol­ecules are linked *via* pairs of C—H⋯O hydrogen bonds, forming inversion dimers. These dimers are linked by π–π inter­actions between the furan rings of neighbouring mol­ecules [centroid–centroid distance = 3.701 (2) Å] into supra­molecular chains running along the *a*-axis direction.

## Related literature   

For background information and the crystal structures of related compounds, see: Choi *et al.* (2008 ▶, 2012*a* ▶,*b* ▶).
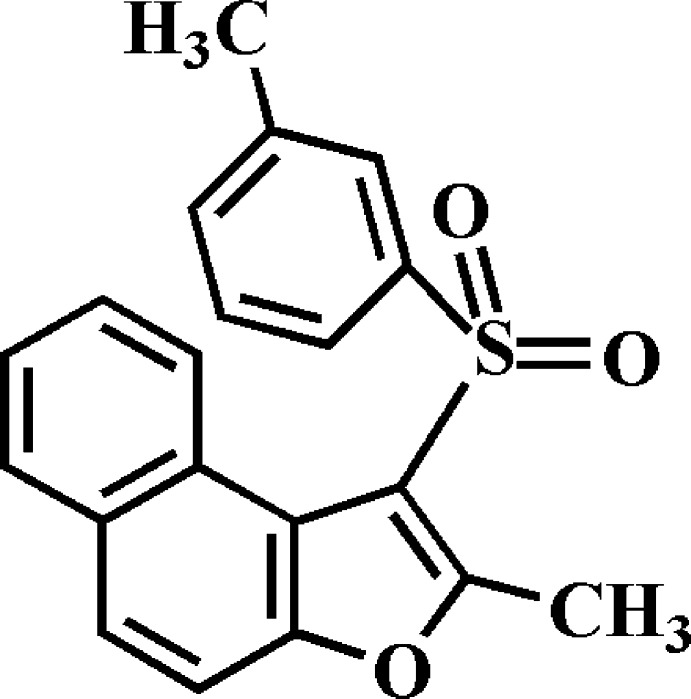


## Experimental   

### 

#### Crystal data  

C_20_H_16_O_3_S
*M* *_r_* = 336.39
Monoclinic, 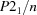

*a* = 11.1667 (2) Å
*b* = 7.7400 (1) Å
*c* = 18.4736 (3) Å
β = 98.683 (1)°
*V* = 1578.38 (4) Å^3^
*Z* = 4
Mo *K*α radiation
μ = 0.22 mm^−1^
*T* = 173 K
0.51 × 0.25 × 0.21 mm

#### Data collection  

Bruker SMART APEXII CCD diffractometer
Absorption correction: multi-scan (*SADABS*; Bruker, 2009 ▶) *T*_min_ = 0.697, *T*_max_ = 0.746
15253 measured reflections
3946 independent reflections
3390 reflections with *I* > 2σ(*I*)
*R*_int_ = 0.026

#### Refinement  

*R*[*F* ^2^ > 2σ(*F* ^2^)] = 0.042
*wR*(*F* ^2^) = 0.117
*S* = 1.05
3946 reflections
219 parameters
H-atom parameters constrained
Δρ_max_ = 0.31 e Å^−3^
Δρ_min_ = −0.52 e Å^−3^

### 

Data collection: *APEX2* (Bruker, 2009 ▶); cell refinement: *SAINT* (Bruker, 2009 ▶); data reduction: *SAINT*; program(s) used to solve structure: *SHELXS97* (Sheldrick, 2008 ▶); program(s) used to refine structure: *SHELXL97* (Sheldrick, 2008 ▶); molecular graphics: *ORTEP-3 for Windows* (Farrugia, 2012 ▶) and *DIAMOND* (Brandenburg, 1998 ▶); software used to prepare material for publication: *SHELXL97*.

## Supplementary Material

Crystal structure: contains datablock(s) I. DOI: 10.1107/S1600536814005157/zq2218sup1.cif

Structure factors: contains datablock(s) I. DOI: 10.1107/S1600536814005157/zq2218Isup2.hkl

Supporting information file. DOI: 10.1107/S1600536814005157/zq2218Isup3.cml

CCDC reference: 990319

Additional supporting information:  crystallographic information; 3D view; checkCIF report

## Figures and Tables

**Table 1:** Hydrogen-bond geometry (Å, °)

*D*—H⋯*A*	*D*—H	H⋯*A*	*D*⋯*A*	*D*—H⋯*A*
C20—H20*B*⋯O2^i^	0.98	2.54	3.507 (2)	171

Symmetry code: (i) 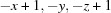.

**Fig. 1.**
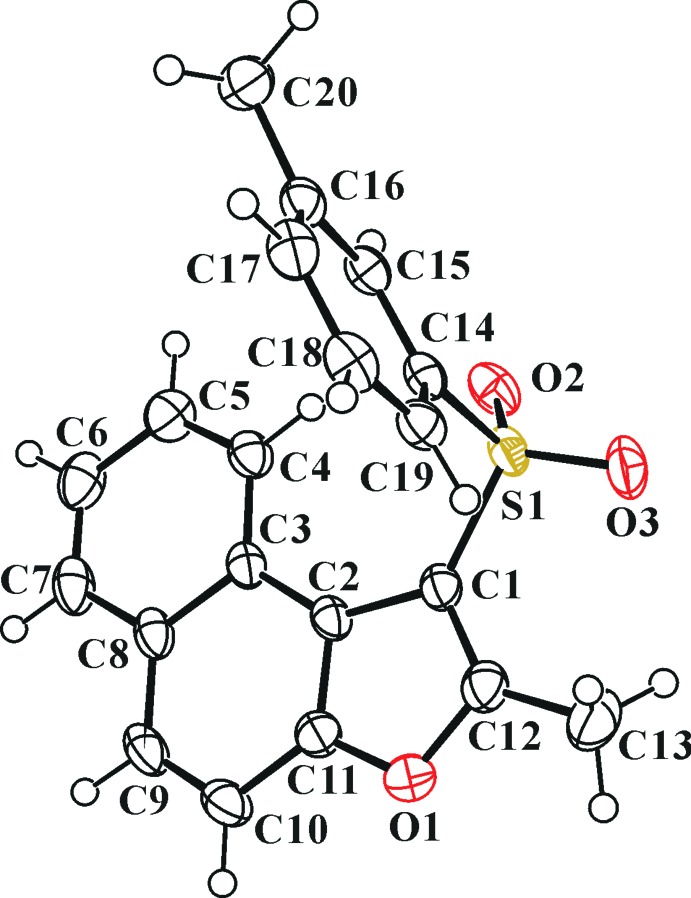
The molecular structure of the title molecule with the atom numbering scheme. The displacement ellipsoids are drawn at the 50% probability level. The hydrogen atoms are presented as small spheres of arbitrary radius.

**Fig. 2.**
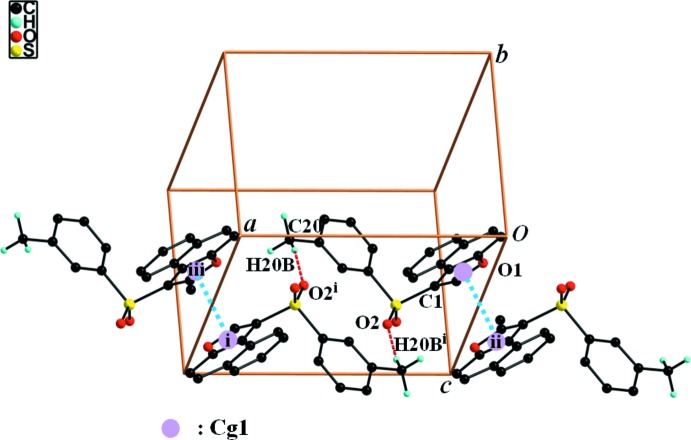
A view of the C–H···O and π–π interactions (dotted lines) in the crystal structure of the title compound. H atoms non-participating in hydrogen-bonding were omitted for clarity. [Symmetry codes: (i) - x + 1, - y, - z + 1; (ii) - x, - y, - z + 1; (iii) x + 1, y, z.]

## supplementary crystallographic information

### 1. Comment

As a part of our continuing study of 2-methylnaphtho[2,1-b]furan derivatives containing phenylsulfonyl (Choi *et al.*, 2008), 4-methylphenylsulfonyl (Choi *et al.*, 2012*a*) and 4-bromophenylsulfonyl (Choi *et al.*, 2012*b*) substituents in 1-position, we report here the crystal structure of the title compound.

In the title molecule (Fig. 1), the naphthofuran unit is essentially planar, with a mean deviation of 0.025 (1) Å from the least-squares plane defined by the thirteen constituent atoms. The 3-methylphenyl ring is essentially planar, with a mean deviation of 0.014 (1) Å from the least-squares plane defined by the six constituent atoms. The dihedral angle formed by the naphthofuran ring system and the 3-methylphenyl ring is 88.56 (2)°. In the crystal structure (Fig. 2), molecules are connected *via* pairs of C–H···O hydrogen bonds (Table 1), forming inversion dimers. These dimers are further packed by π–π interactions between the furan rings of neighbouring molecules, with a Cg1···Cg1^ii^ distance of 3.701 (2) Å and an interplanar distance of 3.503 (2) Å resulting in a slippage of 1.194 (2) Å (Cg1 is the centroid of the C1/C2/C11/O1/C12 furan ring), forming supramolecular chains running along the *a*-axis direction.

### 2. Experimental

3-Chloroperoxybenzoic acid (77%, 538 mg, 2.4 mmol) was added in small portions to a stirred solution of 2-methyl-1-(3-methylphenylsulfanyl)naphtho[2,1-*b*]furan (334 mg, 1.1 mmol) in dichloromethane (40 mL) at 273 K. After being stirred at room temperature for 10h, the mixture was washed with saturated sodium bicarbonate solution and the organic layer was separated, dried over magnesium sulfate, filtered and concentrated at reduced pressure. The residue was purified by column chromatography (hexane-ethyl acetate, 4:1 v/v) to afford the title compound as a colorless solid [yield 72%, m.p. 424-425 K; *R*_f_ = 0.52 (hexane-ethyl acetate, 4:1 *v*/*v*)]. Single crystals suitable for X-ray diffraction were prepared by slow evaporation of a solution of the title compound in benzene at room temperature.

### 3. Refinement

All H atoms were positioned geometrically and refined using a riding model, with C–H = 0.95 Å for aryl and 0.95 Å for methyl H atoms. *U*_iso_ (H) = 1.2*U*_eq_ (C) for aryl and 1.5*U*_eq_ (C) for methyl H atoms. The positions of methyl hydrogens were optimized using the *SHELXL*-97's command AFIX 137 (Sheldrick, 2008).

### Crystal data

**Table:**

C_20_H_16_O_3_S	*F*(000) = 704
*M**_r_* = 336.39	*D*_x_ = 1.416 Mg m^−^^3^
Monoclinic, *P*2_1_/*n*	Melting point = 424–425 K
Hall symbol: -P 2yn	Mo *K*α radiation, λ = 0.71073 Å
*a* = 11.1667 (2) Å	Cell parameters from 5868 reflections
*b* = 7.7400 (1) Å	θ = 2.2–28.3°
*c* = 18.4736 (3) Å	µ = 0.22 mm^−^^1^
β = 98.683 (1)°	*T* = 173 K
*V* = 1578.38 (4) Å^3^	Block, colourless
*Z* = 4	0.51 × 0.25 × 0.21 mm

### Data collection

**Table:**

Bruker SMART APEXII CCD diffractometer	3946 independent reflections
Radiation source: rotating anode	3390 reflections with *I* > 2σ(*I*)
Graphite multilayer monochromator	*R*_int_ = 0.026
Detector resolution: 10.0 pixels mm^-1^	θ_max_ = 28.4°, θ_min_ = 2.0°
φ and ω scans	*h* = −14→14
Absorption correction: multi-scan (*SADABS*; Bruker, 2009)	*k* = −8→10
*T*_min_ = 0.697, *T*_max_ = 0.746	*l* = −24→18
15253 measured reflections	

### Refinement

**Table:**

Refinement on *F*^2^	Primary atom site location: structure-invariant direct methods
Least-squares matrix: full	Secondary atom site location: difference Fourier map
*R*[*F*^2^ > 2σ(*F*^2^)] = 0.042	Hydrogen site location: difference Fourier map
*wR*(*F*^2^) = 0.117	H-atom parameters constrained
*S* = 1.05	*w* = 1/[σ^2^(*F*_o_^2^) + (0.0606*P*)^2^ + 0.6579*P*] where *P* = (*F*_o_^2^ + 2*F*_c_^2^)/3
3946 reflections	(Δ/σ)_max_ = 0.003
219 parameters	Δρ_max_ = 0.31 e Å^−^^3^
0 restraints	Δρ_min_ = −0.52 e Å^−^^3^

### Special details

**Table:**

Geometry. All esds (except the esd in the dihedral angle between two l.s. planes) are estimated using the full covariance matrix. The cell esds are taken into account individually in the estimation of esds in distances, angles and torsion angles; correlations between esds in cell parameters are only used when they are defined by crystal symmetry. An approximate (isotropic) treatment of cell esds is used for estimating esds involving l.s. planes.
Refinement. Refinement of F^2^ against ALL reflections. The weighted R-factor wR and goodness of fit S are based on F^2^, conventional R-factors R are based on F, with F set to zero for negative F^2^. The threshold expression of F^2^ > 2sigma(F^2^) is used only for calculating R-factors(gt) etc. and is not relevant to the choice of reflections for refinement. R-factors based on F^2^ are statistically about twice as large as those based on F, and R- factors based on ALL data will be even larger.

### Fractional atomic coordinates and isotropic or equivalent isotropic displacement parameters (Å^2^)

**Table:**

	*x*	*y*	*z*	*U*_iso_*/*U*_eq_	
S1	0.28214 (3)	0.08312 (5)	0.611416 (19)	0.02882 (12)	
O1	−0.04251 (10)	0.26951 (15)	0.55227 (6)	0.0352 (3)	
O2	0.33118 (11)	−0.05146 (14)	0.57127 (6)	0.0358 (3)	
O3	0.26812 (12)	0.04900 (17)	0.68617 (6)	0.0421 (3)	
C1	0.14243 (13)	0.1526 (2)	0.56462 (8)	0.0277 (3)	
C2	0.10413 (13)	0.18600 (18)	0.48678 (8)	0.0262 (3)	
C3	0.14969 (13)	0.16201 (19)	0.41891 (8)	0.0265 (3)	
C4	0.26063 (14)	0.0823 (2)	0.41147 (8)	0.0314 (3)	
H4	0.3118	0.0418	0.4538	0.038*	
C5	0.29601 (17)	0.0623 (2)	0.34395 (9)	0.0385 (4)	
H5	0.3706	0.0065	0.3401	0.046*	
C6	0.22342 (17)	0.1234 (3)	0.28055 (9)	0.0416 (4)	
H6	0.2493	0.1107	0.2342	0.050*	
C7	0.11597 (16)	0.2006 (2)	0.28576 (9)	0.0393 (4)	
H7	0.0673	0.2417	0.2425	0.047*	
C8	0.07448 (14)	0.2216 (2)	0.35393 (8)	0.0311 (3)	
C9	−0.04140 (15)	0.2957 (2)	0.35690 (9)	0.0375 (4)	
H9	−0.0893	0.3333	0.3128	0.045*	
C10	−0.08502 (14)	0.3139 (2)	0.42102 (10)	0.0368 (4)	
H10	−0.1627	0.3620	0.4231	0.044*	
C11	−0.00957 (14)	0.2578 (2)	0.48414 (8)	0.0303 (3)	
C12	0.05076 (14)	0.2062 (2)	0.60067 (9)	0.0331 (3)	
C13	0.02986 (18)	0.2145 (3)	0.67799 (10)	0.0458 (4)	
H13A	0.0765	0.3104	0.7028	0.069*	
H13B	0.0558	0.1058	0.7027	0.069*	
H13C	−0.0566	0.2327	0.6795	0.069*	
C14	0.37544 (13)	0.26733 (19)	0.61112 (7)	0.0259 (3)	
C15	0.48014 (13)	0.25965 (19)	0.57933 (8)	0.0272 (3)	
H15	0.4995	0.1573	0.5552	0.033*	
C16	0.55711 (14)	0.4026 (2)	0.58282 (8)	0.0298 (3)	
C17	0.52185 (16)	0.5523 (2)	0.61525 (9)	0.0343 (3)	
H17	0.5717	0.6520	0.6165	0.041*	
C18	0.41605 (16)	0.5603 (2)	0.64579 (8)	0.0332 (3)	
H18	0.3937	0.6652	0.6669	0.040*	
C19	0.34312 (14)	0.4162 (2)	0.64559 (8)	0.0301 (3)	
H19	0.2725	0.4187	0.6685	0.036*	
C20	0.67508 (15)	0.3947 (2)	0.55323 (10)	0.0399 (4)	
H20A	0.6927	0.5080	0.5337	0.060*	
H20B	0.6693	0.3083	0.5141	0.060*	
H20C	0.7402	0.3625	0.5926	0.060*	

### Atomic displacement parameters (Å^2^)

**Table:**

	*U*^11^	*U*^22^	*U*^33^	*U*^12^	*U*^13^	*U*^23^
S1	0.0321 (2)	0.0276 (2)	0.02405 (19)	−0.00269 (14)	−0.00458 (14)	0.00574 (13)
O1	0.0277 (5)	0.0399 (6)	0.0380 (6)	−0.0019 (4)	0.0049 (5)	0.0031 (5)
O2	0.0397 (6)	0.0253 (5)	0.0383 (6)	0.0018 (4)	−0.0070 (5)	0.0009 (4)
O3	0.0486 (7)	0.0488 (7)	0.0261 (6)	−0.0086 (6)	−0.0036 (5)	0.0138 (5)
C1	0.0278 (7)	0.0292 (7)	0.0249 (7)	−0.0043 (6)	−0.0001 (5)	0.0041 (6)
C2	0.0250 (6)	0.0254 (7)	0.0259 (7)	−0.0049 (5)	−0.0031 (5)	0.0038 (5)
C3	0.0284 (7)	0.0249 (7)	0.0239 (7)	−0.0065 (5)	−0.0027 (5)	0.0027 (5)
C4	0.0324 (8)	0.0325 (8)	0.0276 (7)	−0.0017 (6)	−0.0010 (6)	0.0009 (6)
C5	0.0406 (9)	0.0416 (9)	0.0331 (8)	−0.0031 (7)	0.0052 (7)	−0.0044 (7)
C6	0.0490 (10)	0.0495 (10)	0.0261 (7)	−0.0123 (8)	0.0052 (7)	−0.0019 (7)
C7	0.0470 (10)	0.0426 (9)	0.0246 (7)	−0.0124 (8)	−0.0062 (7)	0.0061 (7)
C8	0.0340 (8)	0.0290 (7)	0.0273 (7)	−0.0086 (6)	−0.0051 (6)	0.0059 (6)
C9	0.0342 (8)	0.0375 (9)	0.0357 (8)	−0.0032 (7)	−0.0114 (6)	0.0107 (7)
C10	0.0270 (7)	0.0365 (9)	0.0436 (9)	−0.0005 (6)	−0.0056 (6)	0.0077 (7)
C11	0.0269 (7)	0.0299 (8)	0.0330 (8)	−0.0045 (6)	0.0013 (6)	0.0035 (6)
C12	0.0318 (8)	0.0357 (8)	0.0313 (8)	−0.0069 (6)	0.0030 (6)	0.0040 (6)
C13	0.0469 (10)	0.0577 (12)	0.0353 (9)	−0.0073 (9)	0.0140 (8)	0.0013 (8)
C14	0.0278 (7)	0.0264 (7)	0.0210 (6)	0.0002 (5)	−0.0038 (5)	0.0018 (5)
C15	0.0304 (7)	0.0265 (7)	0.0230 (6)	0.0022 (6)	−0.0019 (5)	−0.0009 (5)
C16	0.0307 (7)	0.0331 (8)	0.0239 (7)	−0.0017 (6)	−0.0013 (6)	0.0030 (6)
C17	0.0408 (9)	0.0293 (8)	0.0304 (7)	−0.0062 (6)	−0.0021 (6)	0.0008 (6)
C18	0.0418 (9)	0.0277 (8)	0.0277 (7)	0.0031 (6)	−0.0021 (6)	−0.0039 (6)
C19	0.0313 (7)	0.0333 (8)	0.0244 (7)	0.0044 (6)	0.0000 (6)	0.0000 (6)
C20	0.0347 (8)	0.0470 (10)	0.0383 (9)	−0.0043 (7)	0.0067 (7)	0.0022 (7)

### Geometric parameters (Å, º)

**Table:**

S1—O2	1.4344 (12)	C9—H9	0.9500
S1—O3	1.4375 (12)	C10—C11	1.400 (2)
S1—C1	1.7504 (15)	C10—H10	0.9500
S1—C14	1.7664 (15)	C12—C13	1.483 (2)
O1—C12	1.3577 (19)	C13—H13A	0.9800
O1—C11	1.3665 (19)	C13—H13B	0.9800
C1—C12	1.367 (2)	C13—H13C	0.9800
C1—C2	1.4600 (19)	C14—C15	1.387 (2)
C2—C11	1.380 (2)	C14—C19	1.391 (2)
C2—C3	1.435 (2)	C15—C16	1.397 (2)
C3—C4	1.409 (2)	C15—H15	0.9500
C3—C8	1.4334 (19)	C16—C17	1.388 (2)
C4—C5	1.373 (2)	C16—C20	1.502 (2)
C4—H4	0.9500	C17—C18	1.385 (2)
C5—C6	1.402 (2)	C17—H17	0.9500
C5—H5	0.9500	C18—C19	1.381 (2)
C6—C7	1.357 (3)	C18—H18	0.9500
C6—H6	0.9500	C19—H19	0.9500
C7—C8	1.415 (2)	C20—H20A	0.9800
C7—H7	0.9500	C20—H20B	0.9800
C8—C9	1.424 (2)	C20—H20C	0.9800
C9—C10	1.354 (2)		
			
O2—S1—O3	117.96 (8)	O1—C11—C2	111.60 (13)
O2—S1—C1	110.44 (7)	O1—C11—C10	122.32 (14)
O3—S1—C1	108.22 (7)	C2—C11—C10	126.08 (15)
O2—S1—C14	108.25 (7)	O1—C12—C1	110.18 (13)
O3—S1—C14	107.53 (7)	O1—C12—C13	113.71 (14)
C1—S1—C14	103.44 (7)	C1—C12—C13	136.10 (15)
C12—O1—C11	107.24 (12)	C12—C13—H13A	109.5
C12—C1—C2	107.26 (13)	C12—C13—H13B	109.5
C12—C1—S1	121.98 (12)	H13A—C13—H13B	109.5
C2—C1—S1	130.36 (12)	C12—C13—H13C	109.5
C11—C2—C3	117.84 (13)	H13A—C13—H13C	109.5
C11—C2—C1	103.73 (13)	H13B—C13—H13C	109.5
C3—C2—C1	138.43 (14)	C15—C14—C19	121.55 (14)
C4—C3—C8	118.15 (14)	C15—C14—S1	120.14 (11)
C4—C3—C2	125.10 (13)	C19—C14—S1	118.26 (12)
C8—C3—C2	116.74 (14)	C14—C15—C16	119.92 (14)
C5—C4—C3	121.06 (15)	C14—C15—H15	120.0
C5—C4—H4	119.5	C16—C15—H15	120.0
C3—C4—H4	119.5	C17—C16—C15	117.93 (14)
C4—C5—C6	120.78 (17)	C17—C16—C20	120.87 (15)
C4—C5—H5	119.6	C15—C16—C20	121.20 (15)
C6—C5—H5	119.6	C18—C17—C16	121.89 (15)
C7—C6—C5	119.68 (16)	C18—C17—H17	119.1
C7—C6—H6	120.2	C16—C17—H17	119.1
C5—C6—H6	120.2	C19—C18—C17	120.11 (15)
C6—C7—C8	121.69 (15)	C19—C18—H18	119.9
C6—C7—H7	119.2	C17—C18—H18	119.9
C8—C7—H7	119.2	C18—C19—C14	118.48 (14)
C7—C8—C9	120.12 (14)	C18—C19—H19	120.8
C7—C8—C3	118.63 (15)	C14—C19—H19	120.8
C9—C8—C3	121.22 (15)	C16—C20—H20A	109.5
C10—C9—C8	121.62 (14)	C16—C20—H20B	109.5
C10—C9—H9	119.2	H20A—C20—H20B	109.5
C8—C9—H9	119.2	C16—C20—H20C	109.5
C9—C10—C11	116.46 (15)	H20A—C20—H20C	109.5
C9—C10—H10	121.8	H20B—C20—H20C	109.5
C11—C10—H10	121.8		
			
O2—S1—C1—C12	146.07 (13)	C12—O1—C11—C10	−179.39 (15)
O3—S1—C1—C12	15.58 (16)	C3—C2—C11—O1	178.61 (12)
C14—S1—C1—C12	−98.29 (14)	C1—C2—C11—O1	−0.30 (16)
O2—S1—C1—C2	−42.19 (16)	C3—C2—C11—C10	−1.4 (2)
O3—S1—C1—C2	−172.68 (14)	C1—C2—C11—C10	179.63 (15)
C14—S1—C1—C2	73.45 (15)	C9—C10—C11—O1	179.74 (14)
C12—C1—C2—C11	−0.05 (17)	C9—C10—C11—C2	−0.2 (2)
S1—C1—C2—C11	−172.72 (12)	C11—O1—C12—C1	−0.58 (17)
C12—C1—C2—C3	−178.61 (17)	C11—O1—C12—C13	178.46 (14)
S1—C1—C2—C3	8.7 (3)	C2—C1—C12—O1	0.39 (18)
C11—C2—C3—C4	−176.38 (14)	S1—C1—C12—O1	173.81 (10)
C1—C2—C3—C4	2.0 (3)	C2—C1—C12—C13	−178.34 (19)
C11—C2—C3—C8	2.4 (2)	S1—C1—C12—C13	−4.9 (3)
C1—C2—C3—C8	−179.15 (16)	O2—S1—C14—C15	−3.80 (13)
C8—C3—C4—C5	0.1 (2)	O3—S1—C14—C15	124.65 (12)
C2—C3—C4—C5	178.88 (14)	C1—S1—C14—C15	−120.98 (12)
C3—C4—C5—C6	1.0 (3)	O2—S1—C14—C19	178.42 (11)
C4—C5—C6—C7	−1.1 (3)	O3—S1—C14—C19	−53.14 (13)
C5—C6—C7—C8	−0.1 (3)	C1—S1—C14—C19	61.23 (12)
C6—C7—C8—C9	−176.92 (16)	C19—C14—C15—C16	1.3 (2)
C6—C7—C8—C3	1.1 (2)	S1—C14—C15—C16	−176.43 (10)
C4—C3—C8—C7	−1.1 (2)	C14—C15—C16—C17	−3.3 (2)
C2—C3—C8—C7	179.96 (13)	C14—C15—C16—C20	176.02 (14)
C4—C3—C8—C9	176.90 (14)	C15—C16—C17—C18	2.2 (2)
C2—C3—C8—C9	−2.0 (2)	C20—C16—C17—C18	−177.12 (15)
C7—C8—C9—C10	178.41 (15)	C16—C17—C18—C19	1.0 (2)
C3—C8—C9—C10	0.4 (2)	C17—C18—C19—C14	−3.1 (2)
C8—C9—C10—C11	0.7 (2)	C15—C14—C19—C18	2.0 (2)
C12—O1—C11—C2	0.55 (17)	S1—C14—C19—C18	179.72 (11)

### Hydrogen-bond geometry (Å, º)

**Table:**

*D*—H···*A*	*D*—H	H···*A*	*D*···*A*	*D*—H···*A*
C20—H20*B*···O2^i^	0.98	2.54	3.507 (2)	171

Symmetry code: (i) −*x*+1, −*y*, −*z*+1.
